# ATG9A is an essential host factor for parechovirus RNA replication

**DOI:** 10.1128/jvi.00632-26

**Published:** 2026-06-26

**Authors:** You Li, Lorellin A. Durnell-Bettis, Fahmida Alam, Adriana E. Golding, Juan S. Bonifacino, Matthew R. Vogt

**Affiliations:** 1 Department of Pediatrics, The University of North Carolina at Chapel Hill 549964, Chapel Hill, North Carolina, USA; 2 Department of Microbiology and Immunology, The University of North Carolina at Chapel Hill 318275, Chapel Hill, North Carolina, USA; 3 Division of Neuroscience and Cellular Structure, Eunice Kennedy Shriver National Institute of Child Health and Human Development, National Institutes of Health 2511, Bethesda, Maryland, USA; The University of Texas Southwestern Medical Center, Dallas, Texas, USA

**Keywords:** parechovirus, picornavirus, autophagy, ATG9A, ATG2, Golgi

## Abstract

Parechoviruses (PeVs) are single-stranded, positive-sense RNA viruses in the *Picornaviridae* family. Although most genotypes are not typically associated with severe disease, PeV-A3 has emerged as an important cause of sepsis and meningoencephalitis in newborns. Despite its clinical relevance, the life cycle and pathogenesis of PeV-A3 remain poorly understood. To identify critical host factors for PeV-A3 replication, we performed a genome-wide CRISPR screen. Our results reveal a set of Golgi-localized proteins important for viral infection, among which the lipid scramblase ATG9A (autophagy-related protein 9A) was determined to be essential across different PeV genotypes. Notably, this requirement appears to be independent of canonical autophagy. We further demonstrate that ATG9A is necessary for viral RNA replication, but not entry or translation, and co-localizes with double-stranded RNA, a marker of viral replication organelles (ROs) during infection. ATG2, a lipid transfer protein known to interact with ATG9A, is also required for optimal viral replication. Therefore, the ATG2-ATG9A complex may function by delivering key lipids into the ROs to support viral replication.

**IMPORTANCE:**

Parechovirus (PeV) is a widespread but understudied human pathogen capable of causing severe neurologic disease and sepsis in neonates and infants. Through a CRISPR-based screen, we identified key host factors required for PeV-A3 infection and uncovered a novel, critical role of ATG9A in viral RNA replication that is independent of canonical autophagy. These findings provide new insight into the molecular pathogenesis of the virus and establish a foundation for future antiviral therapeutic development.

## INTRODUCTION

Parechoviruses (PeVs) are a genus of small, non-enveloped, single-stranded positive-sense RNA viruses within the family *Picornaviridae*. These common pathogens are widespread yet largely underrecognized. Human-infecting PeVs fall within the A species (PeV-A), which are classified into multiple genotypes. Immunity to the parechovirus A1 (PeV-A1) genotype is essentially ubiquitous by adulthood, and infection is typically associated with mild gastrointestinal and respiratory presentations. However, some genotypes, while also typically innocuous, can cause severe clinical manifestations in neonates and young infants, with PeV-A3 the most prevalent of these (1, 2). Severe disease includes sepsis-like syndrome, encephalitis, and meningitis with detection of viral RNA (vRNA) in the cerebrospinal fluid, indicating the neurotropism of this specific genotype (3). Infections can result in death or long-term neurologic sequelae of developmental delay (4, 5). The molecular determinants of PeV tissue tropism and virulence in early life remain elusive.

Our current knowledge of the PeV lifecycle is largely based on the study of enteroviruses. While closely related to the *Enterovirus* genus, PeVs have distinct characteristics. The PeV VP0 capsid protein is not cleaved into VP4 and VP2 as in enteroviruses (1). Compared to the enterovirus capsid surface that has a deep “canyon” surrounding the fivefold axis that holds a “pocket factor” (often a fatty acid) critical for capsid uncoating, the PeV capsid surface is flatter and shallower, lacking the pronounced canyon and pocket factor (6). These structural differences are believed to influence cell entry, tissue tropism, and pathogenesis. As a result, antivirals designed to target the canyon or the pocket factor of enteroviruses are not effective against PeVs (7). Recently, myeloid-associated differentiation marker (MYADM) was identified as an essential entry factor for multiple PeV genotypes, including PeV-A1 and -A3 (8, 9), highlighting their distinct receptor usage from enteroviruses. Beyond entry, much of the PeV replication cycle remains uncharacterized.

Autophagy is an evolutionarily conserved process essential for maintaining cellular homeostasis. It involves the sequestration of cytoplasmic components—including damaged organelles and misfolded proteins—within double-membraned vesicles known as autophagosomes, and their subsequent delivery to the lysosome for breakdown and recycling. Autophagy is governed by a cohort of autophagy-related (ATG) proteins that coordinate initiation, nucleation, elongation, and fusion of autophagosomes. A hallmark of autophagosomes is the association of the microtubule-associated protein 1A/1B-light chain 3 (LC3) proteins, in which LC3 is lipidated and becomes attached to the growing phagophore membrane, facilitating cargo recruitment (10). Enteroviruses like the polioviruses and coxsackieviruses actively induce the formation of autophagosome-like vesicles (11–16). These LC3-containing double-membraned structures do not mature into degradative autolysosomes but instead serve as scaffolds for virus replication, assembly, and release (17, 18). A recent study suggested poliovirus nonstructural protein 3CD directly interacts with LC3 via a putative LIR (LC3-interaction region) to promote viral egress (19).

In this study, we carried out a genome-wide CRISPR knockout (KO) screen in search of host factors necessary for PeV-A3 infection. We identified a group of Golgi-localized proteins as important for PeV infection, of which ATG9A, a lipid scramblase involved in early autophagosome biogenesis, was one of the top hits. Cells deficient in ATG9A were highly resistant to both PeV-A1 and PeV-A3 infection. This was independent of downstream canonical autophagy processes, as LC3-KO cells remained susceptible to infection. Mechanistic studies indicated that ATG9A is essential for viral RNA replication but not entry or initiation of viral protein translation. Consistent with this, ATG9A co-localized with double-stranded RNA (dsRNA), a marker of viral replication organelles (ROs) in infected cells. Altogether, these results suggest that ATG9A, likely in complex with its interacting partner ATG2, may play a critical role in the formation of viral ROs.

## RESULTS

### CRISPR screen identified PeV-A3 host factors

**Fig 1:**
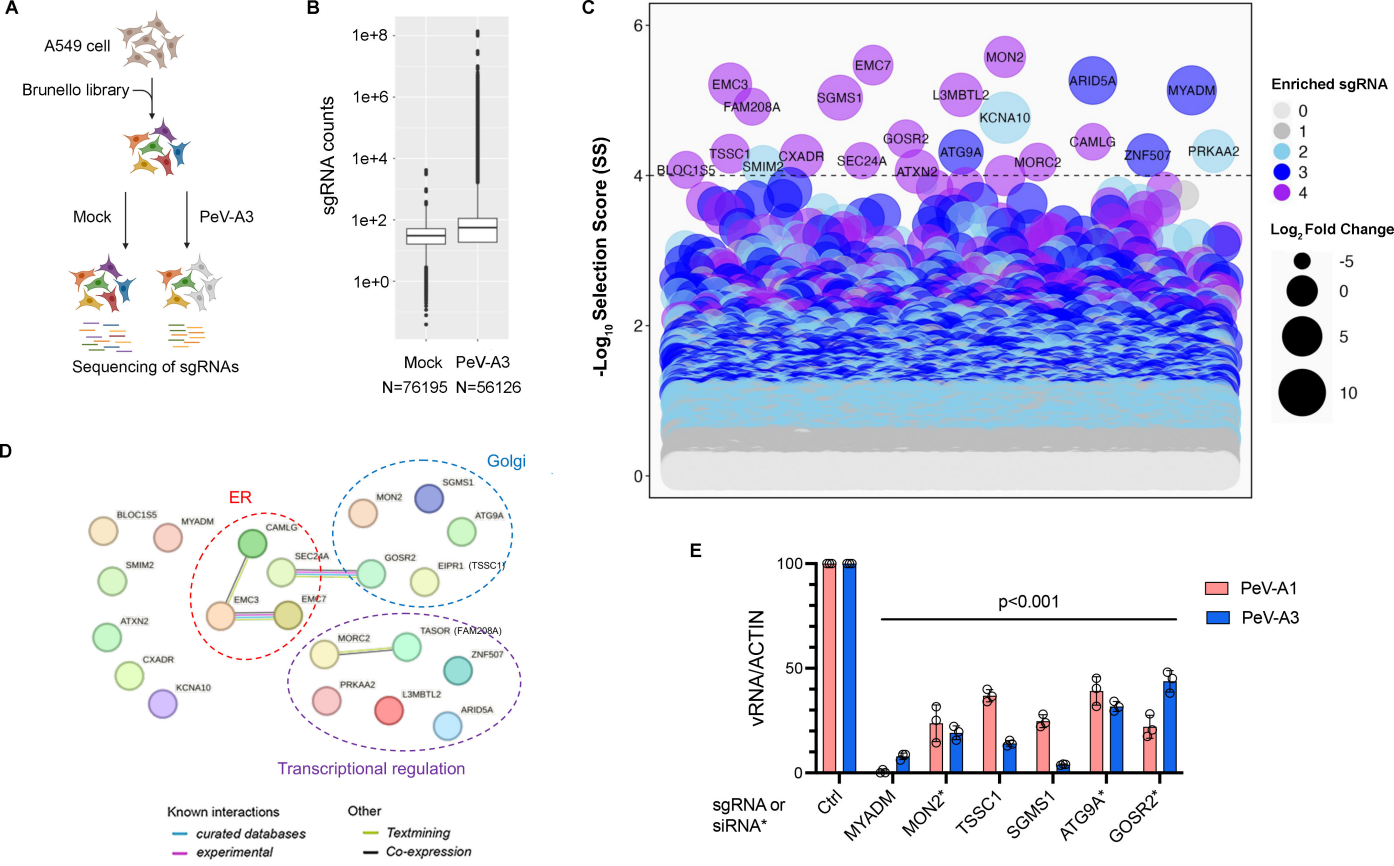
Genome-wide CRISPR screen identifies host factors for PeV-A3 infection. (**A**) Experimental scheme: A549 cells were transduced with the Brunello lentiviral CRISPR library. The mutant cells were infected with PeV-A3 at MOI = 10. Surviving cells were expanded, and sgRNAs were PCR-amplified and sequenced. (**B**) Distribution of sgRNAs in mock- or PeV-A3-selected cells. (**C**) Bubble plot showing enrichment of sgRNAs targeting 19,112 human genes. Bubble size and color reflect log_2_ fold change (LFC) and how many sgRNAs were enriched for each gene, respectively. Placement of bubbles along the *x*-axis is random. *P*-value-based selection scores (SS) were calculated by the MAGeCK algorithm and displayed on a log_10_ scale. Significant hits are labeled as having SS < 0.0001 and LFC > 3. (**D**) STRING analysis of functional associations among proteins encoded by genes comprising the top 21 hits in the screen. (**E**) Quantitative RT-PCR (RT-qPCR) of PeV vRNA in A549 cells with knockdown (siRNA, *) or knockout (sgRNA) of the indicated proteins. Cells were infected at MOI 0.1 for 48 h. vRNA levels were normalized to β-actin RNA, and the levels in control (Ctrl) cells were arbitrarily set to 100. Error bars indicate standard deviation. *P*-values for all hits are <0.001 compared with the Ctrl cells by one-way ANOVA.

To better understand the host factors required for PeV infection, we devised a genome-wide, forward genetic CRISPR (clustered regularly interspaced short palindromic repeats) screen. A549 cells (human lung adenocarcinoma cell line) were transduced with the Brunello lentivirus library expressing single guide RNAs (sgRNAs) targeting 19,114 human genes, each with 4 sgRNAs (20), then subjected to high-multiplicity PeV-A3 virus infection (MOI = 10) (Fig. 1A). Three days post-infection, surviving cells were expanded and sequenced. They were highly enriched in a subset of sgRNA integrants when compared with those in uninfected cells (Fig. 1B). MAGeCK analysis identified 21 candidate host factors with high confidence (selection score ≤1 × 10^−4^ and log_2_ fold change >3) (Fig. 1C). These hits included distinct clusters of functionally related genes, most notably endoplasmic reticulum (ER) and Golgi proteins, and nuclear transcriptional regulators (Fig. 1D). Importantly, we identified the known PeV entry factor MYADM (8, 9) as one of the top hits, validating the CRISPR screen results.

We focused on the hits encoding Golgi proteins, including ATG9A, sphingomyelin synthase 1 (SGMS1), Golgi SNARE complex member 2 (GOSR2), and two proteins involved in endosome-to-Golgi trafficking (regulator of endosome-to-Golgi trafficking [MON2] and tumor-suppressing subtransferable candidate 1 [TSSC1, or EIPR1]). To validate these hits, we depleted each of these proteins in A549 cells using siRNA knockdown or CRISPR KO (Fig. S1). Quantitative RT-PCR (RT-qPCR) confirmed significant reduction of viral RNA in each depleted cell line, and similar effects were observed for PeV-A1 and PeV-A3 (Fig. 1E), indicating these proteins are important for efficient infection of both genotypes. As a positive control, viral RNA was also reduced in MYADM-KO cells.

### ATG9A is an essential parechovirus host factor independent of autophagy

**Fig 2:**
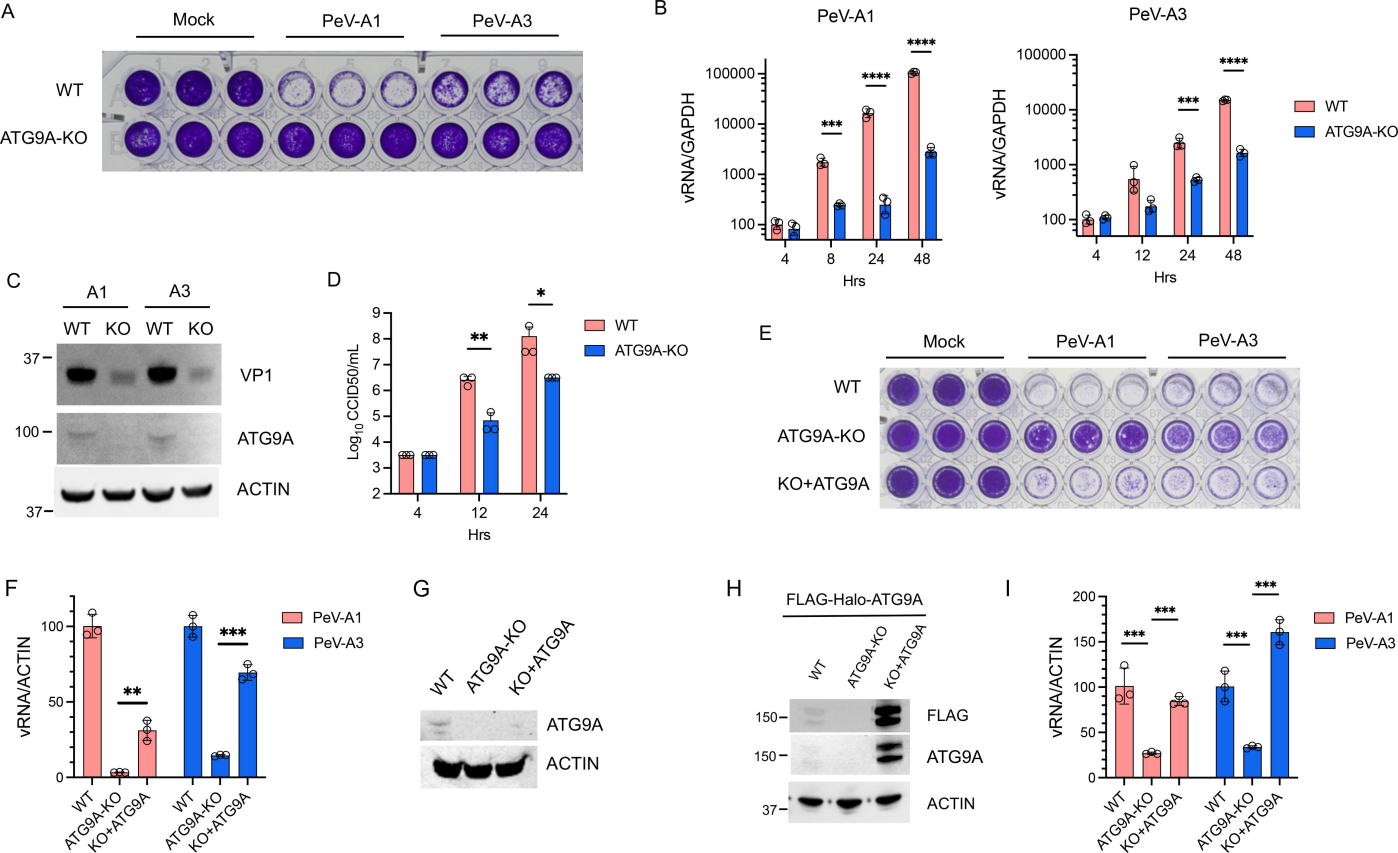
ATG9A is an essential host factor for PeV-A1 and -A3. (**A**) Crystal violet staining of WT or ATG9A-KO HeLa cells infected with PeV-A1 (MOI = 0.1) or -A3 (MOI = 0.5) for 4 days. (**B**) RT-qPCR of vRNA in WT or ATG9A-KO cells infected with PeV-A1 (MOI = 0.1) or -A3 (MOI = 0.1) at indicated time points, normalized to GAPDH RNA. The levels in WT cells at 4 h were arbitrarily set to 100. Error bars indicate standard deviation. ***P < 0.001, ****P < 0.0001 by unpaired *t*-test. (**C**) WT or ATG9A-KO infected with PeV-A1 (MOI = 0.1) or -A3 (MOI = 0.5) for 2 days, immunoblotted for viral VP1, ATG9A, and β-actin. (**D**) Viral titers from WT or ATG9A-KO cells infected with PeV-A1 (MOI = 0.5) at indicated time points, determined by CCID_50_ assay. *P < 0.05, **P < 0.01 by unpaired *t*-test. (**E**) Crystal violet staining of WT HeLa, ATG9A-KO, or ATG9A-KO expressing exogenous ATG9A (KO + ATG9A) infected with PeV-A1 (MOI = 0.1) or -A3 (MOI = 0.5) for 4 days. (**F**) RT-qPCR of vRNA in WT, ATG9A-KO, or ATG9A-reconstituted (KO + ATG9A) HeLa cells, infected with PeV-A1 (MOI = 0.1) or -A3 (MOI = 0.5) for 2 days, and normalized to β-actin RNA. The levels in WT HeLa cells were arbitrarily set to 100. Error bars indicate standard deviation. **P < 0.01, ***P < 0.001 by unpaired *t*-test. (**G**) Immunoblot of ATG9A in cells shown in panels E and F, with β-actin as an internal control. (**H**) U2OS cells in which ATG9A is fused with FLAG-Halo tag at the endogenous locus, where ATG9A is knocked out (ATG9A-KO), and then added back (KO + ATG9A), immunoblotted for FLAG, ATG9A, and β-actin. (**I**) RT-qPCR of vRNA in U2OS cells shown in panel H, infected with PeV-A1 (MOI = 0.1) or -A3 (MOI = 0.5) for 24 h, and normalized to β-actin RNA. The levels in WT cells were arbitrarily set to 100. Error bars indicate standard deviation. ***P < 0.001 by unpaired *t*-test.

ATG9 is the only transmembrane protein in the autophagy pathway and is proposed to be a lipid scramblase that distributes phospholipids between the outer and inner leaflets of autophagosomes (21–24). It traffics between the *trans*-Golgi network (TGN) and sites of autophagosome formation associated with the ER (25). Mammals have two homologous genes, ATG9A and ATG9B. While ATG9B expression is restricted to the placenta and pituitary gland, ATG9A is ubiquitously expressed (26). To further understand the role of ATG9A in PeV infection, we utilized previously described ATG9A-KO HeLa cells (27). HeLa cells were infected with PeV-A1 and -A3, causing substantial cell death (Fig. 2A). In contrast, ATG9A-KO cells were strikingly resistant to viral infection, with little cell death at 4 days post-infection (Fig. 2A). We performed a time course of PeV infection and measured viral RNA levels by RT-qPCR (Fig. 2B). Notably, PeV-A3 replicates much more slowly than PeV-A1. Nonetheless, both genotypes show severely diminished viral replication in ATG9A-KO cells, with >10-fold reduction of viral RNA levels at 24 h and 48 h post-infection. Immunoblots confirmed the depletion of ATG9A protein and showed substantially diminished viral protein levels in the infected ATG9A-KO cells (Fig. 2C). Consistently, viral titers were reduced ~10-fold in ATG9A-KO cells (Fig. 2D). We observed the same phenotype using a different clone of ATG9A-KO HeLa (Fig. 2E and F) (28). Importantly, lentiviral transduction of the ATG9A-KO cells with a construct encoding ATG9A partially rescued virus infection (Fig. 2E and F), despite the low expression level of exogenous ATG9A protein (Fig. 2G). To further confirm the function of ATG9A in PeV infection, we obtained U2OS cells in which a FLAG-Halo tag is fused to ATG9A at the endogenous locus (FLAG-Halo-ATG9A) (29). ATG9A is knocked out in this cell line (ATG9A-KO) and then reconstituted by AAV-mediated expression of FLAG-Halo-ATG9A (KO + ATG9A) (Fig. 2H) (29). Upon PeV infection, viral RNA levels were substantially reduced in the ATG9A-KO cells, and fully restored in ATG9A-reconstituted cells (Fig. 2I). Altogether, these data confirm an essential role of ATG9A in PeV infection.

**Fig 3:**
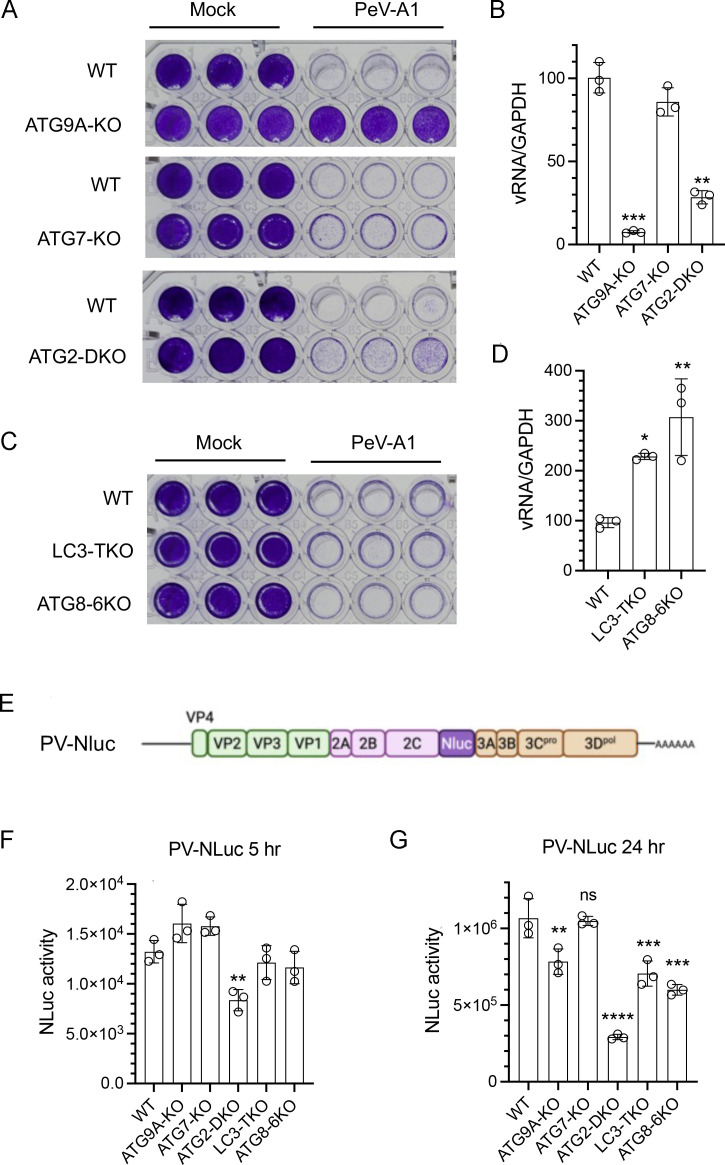
Canonical autophagy pathway is not required for parechovirus infection. (**A**) Crystal violet staining of WT, ATG9A-KO, ATG7-KO, and ATG2-DKO HeLa cells infected with PeV-A1 (MOI = 0.1) for 4 days. (**B**) RT-qPCR of vRNA in indicated cells infected with PeV-A1 (MOI = 0.1) for 2 days, normalized to GAPDH RNA. (**C**) Crystal violet staining of WT, LC3-TKO, and ATG8-6KO HeLa cells infected with PeV-A1 (MOI = 0.1) for 4 days. (**D**) RT-qPCR of viral RNA in indicated cells infected with PeV-A1 (MOI = 0.1) for 2 days, normalized to GAPDH RNA. For panels B and D, the levels in WT cells were arbitrarily set to 100. Error bars indicate standard deviation, and *P < 0.05, **P < 0.01, ***P < 0.001 as compared to WT by one-way ANOVA. (**E**) Schematic depicting the poliovirus 1-nanoluciferase reporter virus (PV-Nluc). HeLa cells were infected with PV-Nluc virus, and luciferase activity was measured at 5 h (**F**) or 24 h (**G**) post-infection. **P < 0.01, ***P < 0.001, and ****P < 0.0001 by one-way ANOVA. ns, not significant.

Autophagy has been demonstrated to be important for enterovirus infection, during which the virus engages LC3-containing autophagosome membranes to promote virus replication, assembly, and spread (14, 15, 17, 19). We tested whether other proteins in the autophagy pathway are involved in PeV infection. We first tested ATG2, which is a lipid transporter that interacts with ATG9A. It has two isoforms, ATG2A and ATG2B (24, 30). Interestingly, PeV viral infection was significantly suppressed in cells deficient in both ATG2A and ATG2B (double knockout, DKO) (27), albeit to a lesser extent than in ATG9A-KO cells (Fig. 3A and B). We also tested ATG7, which is essential for the lipidation of LC3 proteins, a hallmark of the autophagy process. However, knockout of ATG7 had no effect on PeV infection (Fig. 3A and B), suggesting downstream canonical autophagy processes are not important. To further confirm this finding, we used LC3-TKO cells (31) that are depleted of the three LC3 paralogs (LC3A/B/C) and the ATG8-6KO cells that are depleted of all six ATG8 family proteins (LC3A/B/C and GABARAP/L1/L2) (31). We confirmed the knockout of the different autophagy proteins by immunoblots (Fig. S2A and C). Knockout of these proteins did not significantly affect cell proliferation (Fig. S2B and D), except for the ATG2-DKO cells, which showed slightly slower growth. Neither cell line suppressed PeV infection (Fig. 3C and D). In contrast to enteroviruses (32), PeV viral RNA levels were significantly increased in both cell lines (Fig. 3D), suggesting downstream autophagy processes are inhibitory for PeV infection.

To directly compare with enteroviruses, we tested poliovirus replication in these KO cells. We utilized a recombinant reporter poliovirus that expresses nanoluciferase (Fig. 3E). At an early time point (5 h) post-infection, which is within the first replication cycle, only ATG2-DKO showed a significant reduction in luciferase activity (Fig. 3F). At a later time point (24 h) post-infection, corresponding to the completion of several replication cycles, a significant reduction in luciferase activity was also observed for ATG9A-KO, LC3-TKO, and ATG8-6KO cells (Fig. 3G). This result was consistent with the known role of secretory autophagy in poliovirus assembly and release (19, 33, 34), and reveals a substantial difference in the requirement of autophagic factors between PeVs and enteroviruses.

### ATG9A is required for viral RNA replication

**Fig 4:**
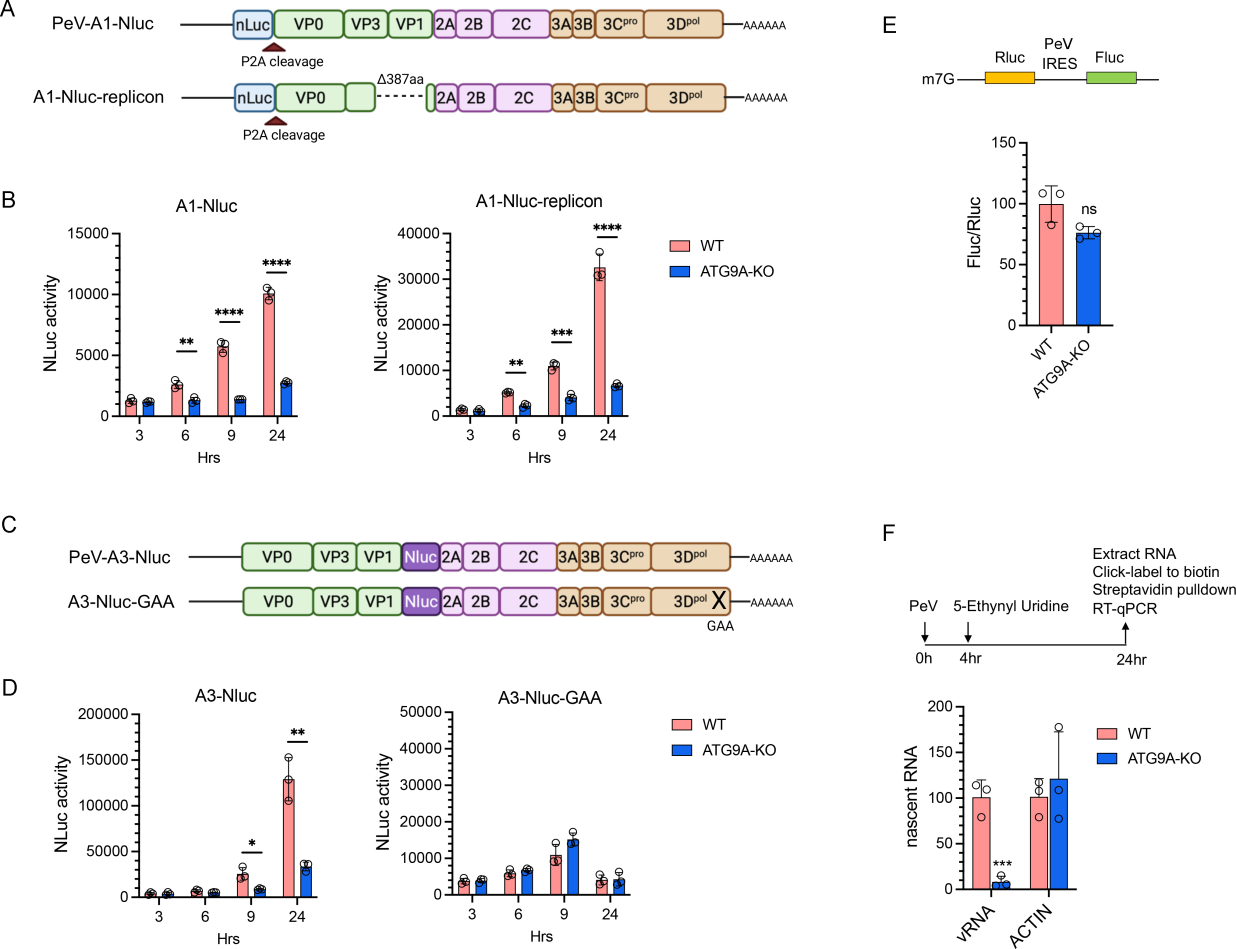
ATG9A is required for viral RNA replication. (**A**) Schematic depicting PeV-A1 RNA containing a nanoluciferase (Nluc) reporter (PeV-A1-Nluc) and the replicon RNA (A1-Nluc-replicon) with partial deletion of VP3 and VP1. (**B**) WT and ATG9A-KO HeLa cells were transfected with PeV-A1-Nluc or A1-Nluc-replicon RNA. Nluc activity was measured at 3, 6, 9, and 24 h post-transfection. **P < 0.01, ***P < 0.001, and ****P < 0.0001 by one-way ANOVA. (**C**) Schematic depicting PeV-A3 RNA containing a nanoluciferase (Nluc) reporter (PeV-A3-Nluc) and the replication-deficient RNA that contains a GDD-to-GAA mutation in the 3D^pol^ (A3-Nluc-GAA). (**D**) PeV-A3-Nluc or A3-Nluc-GAA RNA was transfected into WT and ATG9A-KO cells. Nluc activity was measured at 3, 6, 9, and 24 h post-transfection. *P < 0.05, **P < 0.01 by one-way ANOVA. (**E**) (Top) Schematic depicting a bicistronic reporter that contains the PeV IRES. (Bottom) The bicistronic reporter construct was transfected into WT or ATG9A-KO cells for 24 h. Firefly (Fluc) and Renilla (Rluc) luciferase activities were measured. IRES activity is calculated as the ratio of Fluc/Rluc. (**F**) Nascent RNA synthesis assay. PeV-A1-infected WT or ATG9A-KO cells were metabolically labeled with 5-ethynyl uridine for 20 h. RNA was isolated, click-labeled with biotin, and captured on streptavidin beads for quantitation of nascent viral and actin RNA. Nascent RNA in WT cells was arbitrarily set to 100. ***P < 0.001 by unpaired *t*-test. ns, not significant.

We next sought to dissect the specific step(s) in the PeV lifecycle that require ATG9A. Previous studies suggest that the first cycle of PeV-A1 RNA replication starts between 4 and 8 h post-infection (35, 36). Our time-course experiment (Fig. 2B) revealed no difference in viral RNA levels at 4 h post-infection between WT and ATG9A-KO cells, indicating that virus entry does not require ATG9A. Next, we transfected *in vitro*-transcribed PeV RNA expressing a nanoluciferase reporter to bypass entry. We used the full-length PeV-A1 genome (PeV-A1-Nluc, [8]) or a PeV-A1 replicon RNA containing a partial deletion of VP3 and VP1 (A1-Nluc-replicon, [8]) (Fig. 4A). For both RNA constructs, the Nluc activity increased substantially in WT cells, while a minimal increase was observed in ATG9A-KO cells (Fig. 4B), indicating a defect in viral RNA replication. We also engineered a PeV-A3 reporter construct (PeV-A3-Nluc) and a replication-deficient mutant (A3-Nluc-GAA) that contains a GDD-to-GAA mutation in the catalytic domain of 3D^pol^ (Fig. 4C). Significantly less Nluc activity was evident in the ATG9A-KO cell for the replication-competent A3-Nluc RNA at 9 h and 24 h post-transfection, while no difference was observed for the A3-Nluc-GAA RNA between WT and ATG9A-KO cells (Fig. 4D). These results strongly suggest ATG9A is crucial for viral RNA replication. Importantly, the Nluc activity at 3 h post-transfection was not different between WT and ATG9A-KO cells (Fig. 4B and D), suggesting that initial viral RNA translation was not affected by ATG9A depletion.

Picornaviruses utilize a 5′ internal ribosome entry site (IRES) to initiate translation. To further rule out a role of ATG9A in viral translation, we used a bicistronic reporter construct in which firefly luciferase (Fluc) expression is directed by the PeV IRES and Renilla luciferase (Rluc) expression is controlled by cap-dependent translation (Fig. 4E). When transfected into WT and ATG9A-KO cells, the Fluc/Rluc ratios were not significantly different (Fig. 4E). This confirmed that PeV IRES activity does not require ATG9A and indicated that the loss of Nluc activity in Fig. 4B and D likely indicated a defect in PeV replication in ATG9A-KO cells. Finally, we devised a nascent RNA synthesis assay by measuring the incorporation of 5-ethynyl uridine (5EU) into newly synthesized RNA (Fig. 4F). ATG9A-KO dramatically reduced (~10-fold) the level of nascent PeV RNA while having no effect on nascent β-actin mRNA synthesis over a 20 h treatment window (Fig. 4D), confirming an essential function of ATG9A in viral RNA replication.

### ATG9A co-localizes with dsRNA in infected cells

Previous studies showed that PeV infection disrupts the cellular Golgi complex (35). We confirmed this finding by staining PeV-infected A549 cells for the *cis*-Golgi marker GM130 (Fig. S3A) or the *trans*-Golgi marker TGN46 (Fig. S3B). In contrast to the typical perinuclear localization noted in uninfected cells, both markers were dispersed in infected cells, which are identified by staining with either viral VP0 protein (Fig. S3A) or dsRNA (a hallmark of viral RNA replication, Fig. S3B). It has been reported that exogenously expressed PeV 3A protein localized to the Golgi (37), and we confirmed this by exogenously expressing HA-tagged 3A (Fig. S3C). Overexpressed 3A largely overlaps with GM130 signal and shows a broader localization beyond Golgi, perhaps in the ER. These data suggest that PeV may disassemble the normal Golgi complex to subvert Golgi components for viral replication.

**Fig 5:**
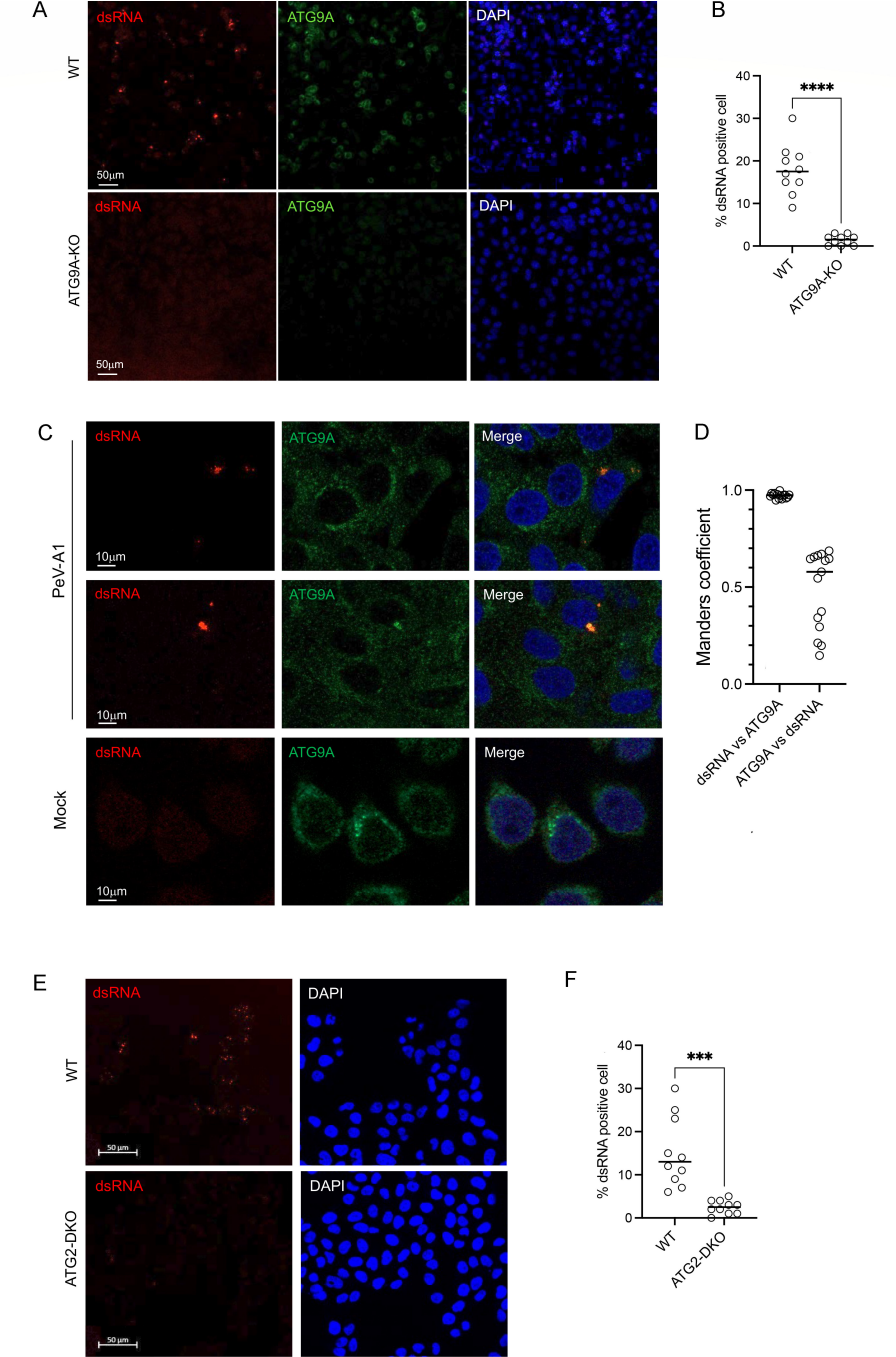
ATG9A co-localizes with dsRNA in infected cells. (**A**) Images showing PeV-A1-infected (MOI = 0.1) WT and ATG9A-KO HeLa cells immunostained for dsRNA (red), ATG9A (green), and DAPI (blue). Scale bar denotes 50 μm. (**B**) Percent of dsRNA-positive cells in PeV-A1-infected WT and ATG9A-KO cells, calculated from 10 images as shown in panel A. ****P < 0.0001 by unpaired *t*-test. (**C**) Mock or PeV-A1-infected WT HeLa immunostained for dsRNA (red), ATG9A (green), and DAPI (blue). Co-localization is shown by yellow color. Scale bar denotes 10 μm. (**D**) Mander’s coefficients of dsRNA vs ATG9A or ATG9A vs dsRNA signals as shown in panel C, *n* = 15 infected cells. (**E**) Images showing PeV-A1-infected (MOI = 0.1) WT and ATG2-DKO HeLa cells labeled with antibodies to dsRNA (red) and DAPI (blue). Scale bar denotes 50 μm. (**F**) Percent of dsRNA-positive cells in PeV-A1-infected WT HeLa and ATG2-DKO cells, calculated from 10 images as shown in panel E. ***P < 0.001 by unpaired *t*-test.

We next examined the localization of ATG9A in infected cells. In accordance with its role in viral RNA replication, very few dsRNA signals could be detected in ATG9A-KO cells compared to WT (Fig. 5A). Quantification of dsRNA-positive cells showed ~10-fold reduction of dsRNA signals in ATG9A-KO (Fig. 5B). Close examination of ATG9A distribution in infected WT HeLa cells revealed substantial colocalization with dsRNA (Fig. 5C). The same was observed in A549 cells with either PeV-A1 or -A3 (Fig. S4). Quantitative analysis revealed that dsRNA was found to almost entirely overlap with endogenous ATG9A (Mander’s overlap coefficient [MOC] >0.9) (Fig. 5D), while the MOCs of ATG9A vs dsRNA were more diverse, ranging from 0.1 to 0.7 (Fig. 5D). This suggests that ATG9A is essentially ubiquitously present at sites of viral RNA replication, but not all ATG9A is engaged in virus replication. dsRNA signal was similarly reduced in ATG2-DKO cells (Fig. 5E and F), consistent with a role of ATG2 in optimal PeV infection (Fig. 3A). Due to the non-specificity of the ATG2 antibody for immunofluorescence studies, we were unable to determine the localization of endogenous ATG2 in infected cells. Taken together, our data suggest that ATG9A, likely alongside ATG2, is recruited to and helps assemble and/or maintain viral ROs during infection.

## DISCUSSION

In this study, we used a genome-wide CRISPR screen to identify key host factors for PeV-A3 infection. Similar screens previously performed with PeV-A1, -A2, and -A3 (8, 9) have also identified host factors, most notably the MYADM protein as a pan-genotype entry factor. In contrast, integrin β6 (ITGB6) was found to be important only for PeV-A1 and -A2 but not -A3 (8). Consistent with these prior studies, our screen found MYADM as one of the top hits (Fig. 1C). ITGB6 sgRNA is also enriched in our screen but does not reach statistical significance (Table S1), consistent with the previous report that integrins are not essential for PeV-A3 entry. In addition, our screen identified many unique host factors compared to the previous screens, including a set of Golgi-localized proteins. Several of these hits (TSSC1, MON2, and GOSR2) are involved in membrane trafficking/vesicular transport. Given the observation that exogenously expressed PeV 3A protein localized to the Golgi (Fig. S3C), these proteins may be involved in transporting viral proteins to the Golgi complex, which then remodel the Golgi membranes to build viral ROs.

Autophagy has emerged as a critical interface between host cells and viral pathogens, exerting both antiviral and proviral effects depending on the virus and cellular context. Autophagy can target viral proteins, genomes, or entire virions for lysosomal degradation, thereby restricting replication of viruses such as Sindbis virus and HIV (38, 39). Conversely, viruses such as enteroviruses and influenza have evolved strategies to co-opt the autophagy machinery to support their life cycles (40). Poliovirus and coxsackievirus B3 infections induce LC3 lipidation and subvert autophagosome-derived membranes for virus replication, assembly, and release. Our data reveal both similarities and distinctions between PeV and poliovirus, two members of the *Picornaviridae* family from different genera, in their utilization of autophagy-related proteins. Both viruses required ATG2 for optimal replication (Fig. 3), but they differed in their dependency on ATG9A. Whereas ATG9A-KO directly suppressed PeV RNA synthesis (Fig. 4), its effect on poliovirus was detectable only at late stages of infection, after completion of the first replication cycle (Fig. 3F and G). This is consistent with previous findings showing that ATG9A deficiency more strongly impairs poliovirus virion production than RNA replication (32). Additionally, LC3-KO inhibited poliovirus at late stages (Fig. 3F and G), but, in contrast, enhanced PeV infection (Fig. 3D). These observations suggest that PeV does not utilize the canonical autophagy pathway, but instead selectively exploits ATG2 and ATG9. In this model, canonical LC3-dependent autophagy would compete for ATG9 availability, thereby restricting PeV replication. Autophagy-independent roles of ATG9 have been described previously, such as lipid droplet mobilization and lysosomal homeostasis (27, 41, 42). Our finding expands the spectrum of ATG9 function that does not rely on the canonical autophagy process.

ATG2 and ATG9 are core components of the autophagy machinery that function at early steps of autophagosome formation. ATG2 acts as a lipid-transfer protein that bridges the endoplasmic reticulum and nascent phagophores (30). ATG9 is the only transmembrane autophagy protein and cycles between the Golgi, endosomes, and autophagosome formation sites (25). It functions as a lipid scramblase which shuffles lipids between the outer and inner leaflets of the membrane (21–24). In canonical autophagy, ATG2 interacts with ATG9, and their coordinated action is essential for maintaining appropriate membrane supply and curvature, enabling membrane expansion during autophagosome biogenesis (24). The co-localization of ATG9 with dsRNA during PeV infection (Fig. 5B) suggests that the virus likely repurposes ATG2- and ATG9-mediated membrane/lipid trafficking to build ROs. How the virus recruits the ATG2-ATG9 complex into ROs remains to be further elucidated. Curiously, the defect of PeV infection in ATG2-DKO is less severe than ATG9-KO (Fig. 3A and B). It is possible that other lipid transporters that interact with ATG9A, such as VPS13 (43), could partially compensate for the role of ATG2 in ATG2-DKO cells.

Given the known function of the ATG2-ATG9 complex, it seems possible that they may support PeV RNA replication by providing essential lipids into the viral ROs. It is well established that ROs of positive-sense RNA viruses, including picornaviruses, flaviviruses, and coronaviruses, are enriched in specific lipids. These lipids not only support the physical structure of ROs but also create a metabolic niche optimal for efficient viral RNA replication. Phosphatidylinositol lipids (e.g., PI4P) are among the best studied for enteroviruses: they hijack host kinase PI4KIIIβ to enrich PI4P at replication sites, creating a lipid microenvironment that recruits viral proteins (44–46). Cholesterol is another key component that helps maintain the tightly curved architecture characteristic of ROs (47, 48). Sphingomyelin has been implicated as an essential lipid for the replication of multiple RNA viruses, and its biosynthetic enzyme SGMS1 has emerged as a critical host factor for infections such as severe fever with thrombocytopenia syndrome virus (SFTSV), SARS-CoV-2, lymphocytic choriomeningitis virus (LCMV), and dengue virus (49). Our data suggest that sphingomyelin is also required for PeV infection, as SGMS1 depletion significantly repressed virus replication (Fig. 1E). In the case of SFTSV, the viral RNA-dependent RNA polymerase (RdRp) could directly bind a specific sphingomyelin species, SM(d18:1/16:1), via a helix-turn-helix motif (49). A similar motif is also present in the RdRps of SARS-CoV-2 and LCMV (49). Although the structure of the PeV RdRp has not yet been experimentally resolved, AlphaFold 3 predicts a similar motif near its C-terminus (data not shown). While the function of sphingomyelin in PeV infection needs to be further studied, it is possible that sphingomyelin facilitates PeV replication by promoting the localization or enrichment of the viral polymerase within ROs. However, sphingomyelin is not a preferred substrate of ATG2 (30), so whether sphingomyelin is transported to ROs by ATG2 or engaged by a different mechanism during PeV infection requires further investigation.

## MATERIALS AND METHODS

### Cells

Vero and A549 cells were obtained from the American Type Culture Collection, maintained in DMEM with 2%–10% fetal bovine serum and 1% penicillin/streptomycin. Cells tested negative for mycoplasma by PCR assay (LookOut Mycoplasma PCR Detection Kit, Sigma). Vero cell overexpression MYADM (Vero-MYADM) and A549 MYADM-KO cells were kindly provided by Dr. Jan Carette (Stanford University, CA) (8). HeLa WT, ATG9A-KO, and ATG2A/B-DKO were previously described (27). LC3-TKO and ATG8-6KO cells were kindly provided by Dr. Richard Youle (NINDS/NIH, Maryland) and Michael Lazarou (Monash University, Melbourne, Australia) (31). ATG9A-KO HeLa cells reconstituted with exogenous ATG9A were described previously (28). U2OS cells expressing FLAG-Halo-tagged ATG9A, and the corresponding ATG9A-KO and ATG9A-addback cells, were provided by Jens Schmidt (Michigan State University) (29). For each modified cell line used, the corresponding WT cell line from which it was derived was used for comparison as obtained from each source laboratory.

### Viruses and plasmids

PeV-A1 Harris strain was obtained from the American Type Culture Collection (#VR-52). PeV-A3 strain US/MO-KC/2014/001 was obtained from BEI Resources (#NR-51187). Viruses were propagated in Vero cells, and viral titers (CCID_50_/mL) were determined in Vero-MYADM cells. The PeV-A1 reporter (PeV-A1-NLuc) and replicon (A1-Nluc-replicon) constructs have been described previously (8). Poliovirus reporter (PV-Nluc) was gifted by Dr. Craig Cameron (University of North Carolina at Chapel Hill), and this reporter virus was propagated in HeLa cells. PeV-A3 reporter (PeV-A3-NLuc) and replication-deficient (A3-NLuc-GAA) constructs were made by assembling *in vitro*-synthesized DNA fragments (Genewiz) using the HiFi DNA Assembly Master Mix (NEB).

### siRNAs and sgRNAs

Smartpool siRNAs targeting human MON2, ATG9A, GOSR2, and a nontargeting control (siCtrl) were purchased from Horizon Discovery. sgRNAs targeting SGMS1 (GACGACCGAGATCATCACTG) and TSSC1 (AGATTTCACCCGCTTGATGG) were synthesized at GenScript and cloned into the lentiCRISPRv2 vector (Addgene, #52961).

### Antibodies

The following antibodies were used in these studies: anti-PeV VP1 (GeneTex, #GTX638649); anti-PeV VP0 (GeneTex, #GTX638759); anti-dsRNA J2 clone (Exalpha, #10010500); anti-β-actin (Sigma, #A2228); anti-ATG9A (Abcam, #ab108338); anti-GM130 (BD Biosciences, #610823); anti-TGN46 (Abcam, #ab50595); anti-MON2 (Thermo Fisher, #A304-814A); anti-SGMS1 (Proteintech, #19050-1-AP); anti-TSSC1 (Thermo Fisher, #PA5-22360); anti-LC3 (Proteintech, #14600-1-AP); anti-GABARAP (Proteintech, #18723-1-AP); anti-ATG2A (Proteintech, #23226-1-AP); anti-ATG2B (Proteintech, #25155-1-AP); anti-ATG7 (Cell Signaling Technology, #8558); and anti-GOSR2 (Proteintech #12095-1-AP).

### Genome-wide CRISPR screen

The Brunello lentiviral pooled library was purchased from Addgene (#73179-LV). A549 cells were transduced with the lentivirus pool at MOI 0.3 and selected by growth in 3 μg/mL puromycin for 1 week. Approximately 4 × 10^7^ puromycin-resistant cells were infected with PeV-A3 virus at an MOI of 10 for 3 days, when greater than 95% of the cells were killed. The surviving cells were expanded for 3–4 weeks, and DNA was isolated by the Quick-DNA Midi Prep Kit (ZYMO Research, #D4075). Amplicons containing sgRNA integrants were amplified through PCR using Q5 Hot Start High-Fidelity DNA Polymerase (New England Biolabs) and the Illumina TruSeq P5 and P7 primers with barcodes and sequencing adapters. PCR products were purified with the AMPure XP reagent (Beckman Coulter) and analyzed on a Qubit 4 fluorometer (Invitrogen), then diluted to 4 nM and pooled for sequencing. Sequencing was carried out on a HiSeq system (Illumina) at Genewiz. The resultant FASTQ files were analyzed using MAGeCK software (50) to identify enriched sgRNA integrants, and a guide enrichment bubble plot was generated with R software version 4.4.

### RT-qPCR quantitation of viral RNA

Total RNA was extracted from cells using the RNeasy Kit (Qiagen) following the manufacturer’s protocol. RT-qPCR was carried out with the iTaq SYBR Green One-Step RT-qPCR Kit (Bio-Rad, #1725151) and the CFX Opus 96 Real-Time PCR System (Bio-Rad). The primers for PeV were 5′-ctggggccaaaagcca-3′ and 5′-ggtaccttctgggcatccttc-3′. Primers for GAPDH were 5′-aatcccatcaccatcttccag-3′ and 5′-aaatgagccccagccttc-3′. Primers for β-actin were 5′-caccattggcaatgagcggttc-3′ and 5′-aggtctttgcggatgtccaggt-3′.

### Luciferase assays

Cells were lysed in 1× passive lysis buffer (Promega, #E1941) for 15 min at room temperature, then lysates were transferred to opaque white 96-well plates (Corning, #3912). NLuc activity was quantified using the NLuc GLOW Assay kit (Nanolight Technology, #325). FLuc and Rluc assays were carried out with the Dual-Glo Luciferase Assay System (Promega, E2920). Luminescence was measured using a BioTek Synergy II multi-mode plate reader (Agilent Technologies).

### Transfections

Viral RNA was transcribed *in vitro* from plasmid DNA using the MEGAscript T7 Transcription Kit (Invitrogen #AM1334). RNA transfections were carried out with the Trans-IT mRNA reagent (Mirus Bio, #MIR 2225), and plasmid transfections with Trans-IT LT1 reagent (Mirus Bio, #MIR 2304) following the manufacturers’ suggested protocols. siRNAs were transfected with Lipofectamine RNAiMAX (Thermo Fisher, #13778030) at 20 nM concentration following the manufacturers’ suggested protocol.

### Crystal violet staining

Cells in 96-well plates were fixed with 1% paraformaldehyde for 15 min at room temperature. Cells were then stained with 1% crystal violet in 20% ethanol for 5 min at room temperature, and excess solution was washed with water.

### Immunoblots

Approximately 10^6^ HeLa or A549 cells were lysed in radioimmunoprecipitation assay buffer (20-188, Millipore) for 20 min on ice and clarified by centrifugation at 14,000 × *g* for 10 min at 4°C. The lysate was mixed with 4× Laemmli buffer, incubated at 95°C for 5 min, then resolved in a 4%–12% gradient SDS-polyacrylamide pre-cast gel (NP0322BOX, Thermo Fisher). Proteins were transferred to a polyvinylidene fluoride membrane by the iBlot2 Gel Transfer Device (Thermo Fisher). Membranes were blocked in Odyssey Blocking Buffer (LI-COR Biosciences) and probed with a 1:1,000 dilution of primary antibodies overnight at 4°C. The membrane was washed with 0.05% Tween-20 and probed with a 1:10,000 dilution of appropriate secondary antibodies conjugated with IRDye 800 or IRDye 680 (LI-COR Biosciences) for 1 h at room temperature. Excess secondary antibodies were removed by washing with 0.05% Tween-20, and protein bands were visualized using an Odyssey Infrared Imaging System (LI-COR Biosciences).

### Nascent RNA synthesis assay

Nascent RNA transcripts were quantified using the Click-iT Nascent RNA Capture Kit (C10365, Thermo Fisher) according to the manufacturer’s protocol with some modifications. HeLa cells were infected with PeV-A1 (MOI = 1) for 4 h, then refed with media containing 0.5 mM 5EU and incubated for an additional 20 h. Total RNA was extracted using the RNeasy Mini Kit (Qiagen). Ten micrograms of total RNA and 0.5 mM biotin azide were used in a copper-catalyzed click reaction to conjugate biotin to 5EU-labeled RNA. Following precipitation, the RNA was dissolved in 50 μL RNase-free water. Biotin-conjugated 5EU-labeled RNA was isolated from 1 μg of total RNA on streptavidin magnetic beads and used as a template for RT-qPCR (see above).

### Confocal immunofluorescence microscopy

Cells infected with PeV for 24 h were visualized in six micro-well glass-bottom plates (Cellvis, Cat. #P06-14-1.5-N). Cells were fixed with 4% paraformaldehyde for 15 min, washed twice with PBS, and permeabilized in 0.2% Triton X-100 for 12 min. Cells were incubated with a blocking solution containing 10% goat serum for 1 h at room temperature. Dishes were then incubated for 1 h at room temperature with primary antibody diluted in PBS with 3% BSA, then washed with PBS containing 0.1% Tween-20, followed by incubation with appropriate secondary antibodies for 1 h. Nuclei were counterstained with DAPI (1 μg/mL) for 5 min at room temperature, and the dishes were then washed three times with PBS. Imaging data were recorded on a laser-scanning confocal Zeiss 980 microscope (Carl Zeiss AG, Oberkochen, Germany) controlled by Zen Blue 3.0 software.

### Statistical analysis

Unless otherwise stated, statistical significance was assessed by unpaired two-sided *t*-test or ANOVA. All statistical calculations were carried out using Prism 10.4.1 software (GraphPad).

## Data Availability

Data described in the paper are available in the figures or supplemental material.

## SUPPLEMENTAL MATERIAL

Supplemental figures jvi.00632-26-s0001.pdf Fig. S1 to S4.

Table S1 jvi.00632-26-s0002.xlsx CRISPR screen hits for parechovirus A3 host factors.

ASM does not own the copyrights to Supplemental Material that may be linked to, or accessed through, an article. The authors have granted ASM a non-exclusive, world-wide license to publish the Supplemental Material files. Please contact the corresponding author directly for reuse.
