# Supplementary material for: ATG9A is an essential host factor for parechovirus RNA replication
Source: J Virol. 2026 Jun 26;100(7):e00632-26. doi: 10.1128/jvi.00632-26 (PMC13386875; doi:10.1128/jvi.00632-26)
Supplement: Supplemental figures — Fig. S1 to S4. [file jvi.00632-26-s0001.pdf]

SUPPLEMENTAL MATERIAL

ATG9A is an essential host factor for parechovirus RNA replication

You Li, Lorellin Durnell, Fahmida Alam, Adriana E. Golding, Juan S. Bonifacino, and Matthew R. Vogt

SUPPLEMENTARY DATA

Supplementary Table S1. CRISPR screen hits for parechovirus A3 host factors.

Supplementary Figures

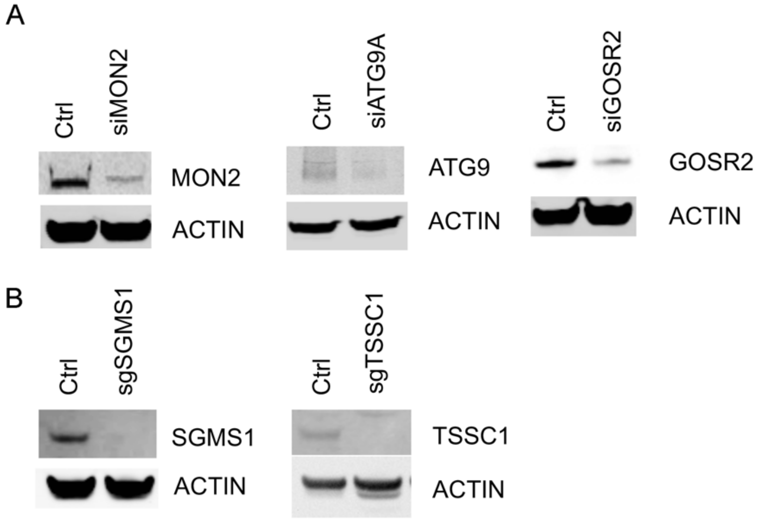

Figure S1. Confirming gene expression knockdown or knockout. (A) Immunoblots of A549 cells transfected with Ctrl siRNA or siRNAs targeting MON2, ATG9A or GOSR2 for knockdown. **(B)** Immunoblots of A549 cells stably transduced with lentiviruses expressing sgRNAs targeting SGMS1 or TSSC1 for knockout. Ctrl: cells expressing Cas9 only.

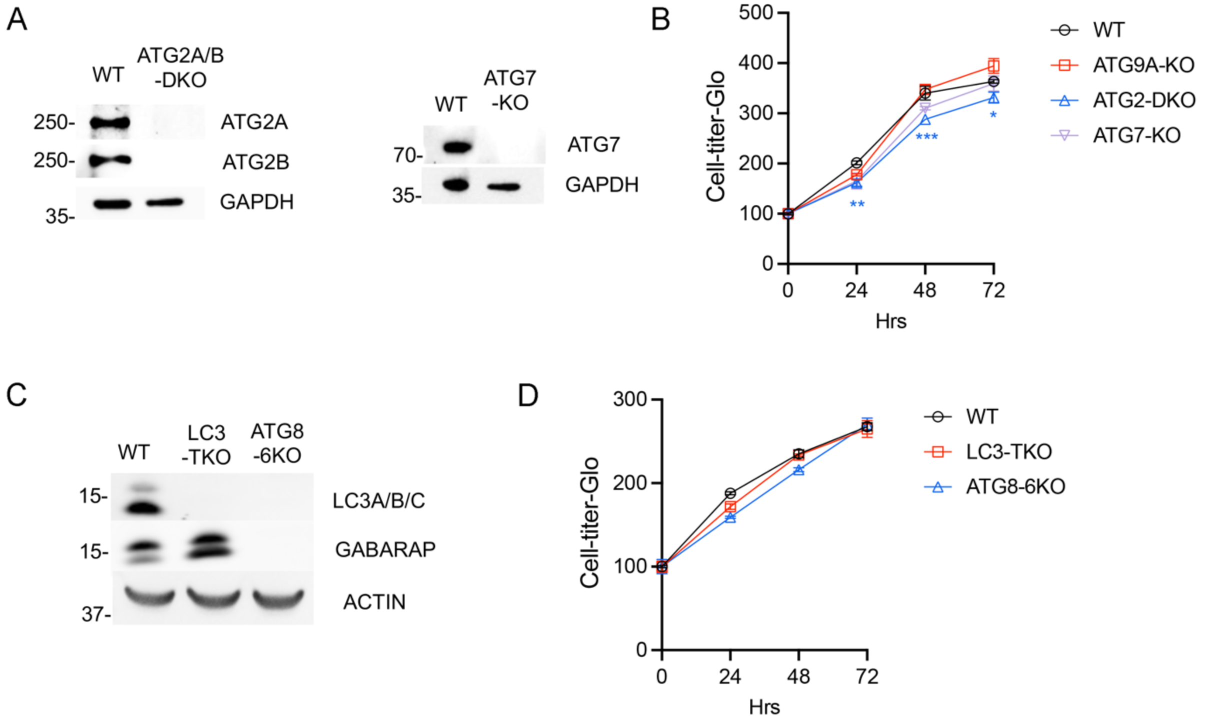

Figure S2. Confirming ATG knockout cell lines. (A) Immunoblots of ATG2A/B (left) and ATG7 (right) in WT and KO HeLa cells, with GAPDH as internal control. (**B**) Growth curve of WT and indicated KO HeLa cells, determined by Cell-titer-Glo assay. Luciferase activity at 0 hr was arbitrarily set to 100. *$p<0.05$, ** $p<0.01$, *** $p<0.001$ by one-way ANOVA. **(C)** Immunoblots of LC3 and GABARAP in WT, LC3-TKO and ATG8-6KO HeLa cells, with β-actin as internal control. (**D**) Growth curve of WT, LC3-TKO and ATG8-6KO HeLa cells, determined by Cell-titer-Glo assay. Luciferase activity at 0 hr was arbitrarily set to 100.

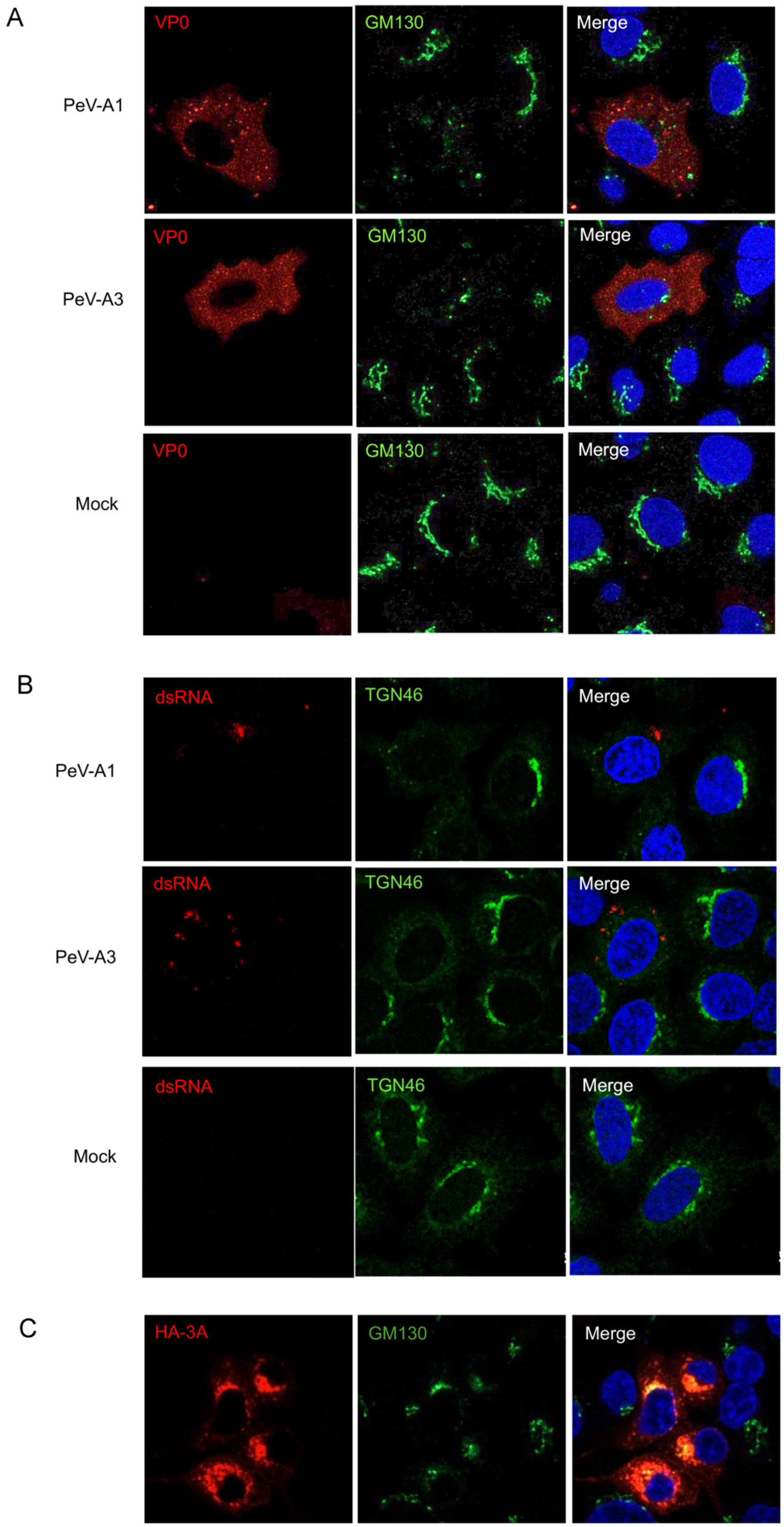
A
VP0
GM130
Merge
PeV-A1
PeV-A3
Mock
B
dsRNA
TGN46
Merge
PeV-A1
PeV-A3
Mock
C
HA-3A
GM130
Merge

Figure S3. PeV infection disrupts Golgi architecture. Mock, PeV-A1 (MOI=0.1), or PeV-A3 (MOI=0.1) infected A549 cells were immunostained for **(A)** VP0 (red) and GM130 (green), or **(B)** dsRNA (red) and TGN46 (green) and both with DAPI (blue). **(C)** A549 cells were transfected with HA-3A, and immunostained for HA (red), GM130 (green) and DAPI (blue).

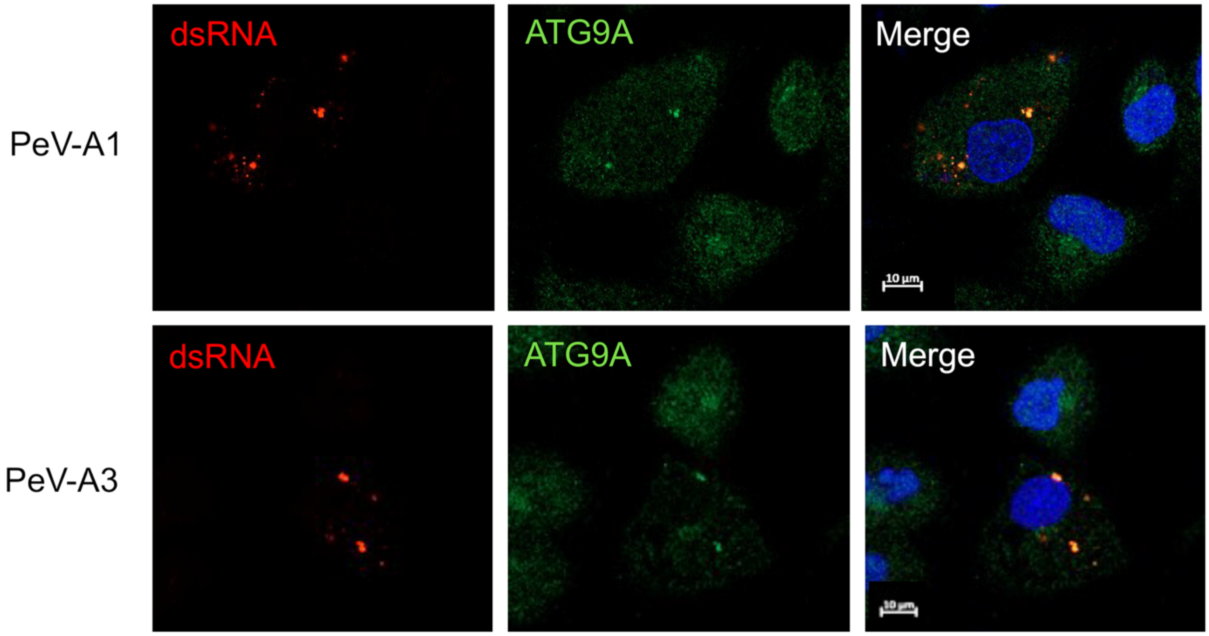

Figure S4. ATG9A co-localizes with dsRNA in infected cell. PeV-A1 or -A3 (MOI=0.1) infected A549 cells were labelled with antibodies to dsRNA (red), ATG9A (green) and DAPI (blue).
